# What Will Be the Impact of the COVID-19 Quarantine on Psychological Distress? Considerations Based on a Systematic Review of Pandemic Outbreaks

**DOI:** 10.3390/healthcare9010101

**Published:** 2021-01-19

**Authors:** Marco Cavicchioli, Roberta Ferrucci, Matteo Guidetti, Maria Paola Canevini, Gabriella Pravettoni, Federica Galli

**Affiliations:** 1 Department of Psychology, University “Vita-Salute San Raffaele”, Via Stamira d’Ancona, 20, 20127 Milan, Italy; cavicchioli.marco@hsr.it; 2 Unit of Clinical Psychology and Psychotherapy, San Raffaele-Turro Hospital Ville Turro Site, Via Stamira d’Ancona, 20, 20127 Milan, Italy; 3 Asst SS.Paolo e Carlo, S.Paolo Hospital, 20142 Milan, Italy; roberta.ferrucci@unimi.it (R.F.); mariapaola.canevini@unimi.it (M.P.C.); 4 Department of Health Science, University of Milan, 20142 Milan, Italy; 5 Aldo Ravelli Center, Department of Health Science, University of Milan, 20142 Milan, Italy; matteo.guidetti@unimi.it; 6 Fondazione IRCCS Ca’ Granda, Ospedale Maggiore Policlinico, 20122 Milan, Italy; 7 Department of Electronics, Information and Bioengineering, Polytechnic University of Milan, 20122 Milano, Italy; 8 European Institute of Oncology, 20141 Milan, Italy; gabriella.pravettoni@unimi.it; 9 Department of Oncology and Hemato-Oncology, University of Milan, 20122 Milan, Italy

**Keywords:** COVID-19, psychological distress, anxiety, depression, PTSD

## Abstract

*Background*: The novel coronavirus (SARS-CoV-2) and related syndrome (COVID-19) has led to worldwide measures with severe consequences for millions of people. In the light of the psychopathological consequences of restrictive measures detected during previous outbreaks, a systematic review was carried out to provide an evidence-based assessment of possible effects of the current COVID-19 quarantine on mental health. *Methods*: This review included studies that assessed mental health indexes (e.g., overall psychological distress, depressive and PTSD symptoms) during and after quarantine periods adopted to management different outbreaks (e.g., COVID-19, SARS, MERS). *Results*: Twenty-one independent studies were included for a total of 82,312 subjects. At least 20% of people exposed to restrictive measures for the management of pandemic infections reported clinically significant levels of psychological distress, especially PTSD (21%) and depressive (22.69%) symptoms. Overall, original studies highlighted relevant methodological limitations. *Conclusions*: Nowadays, almost one out of every five people is at risk of development of clinically significant psychological distress. Further research on mental health after the current COVID-19 quarantine measures is warranted.

## 1. Introduction

On 12 March 2020, the World Health Organization (WHO) declared that the 2019 novel coronavirus (SARS-CoV-2) and related syndrome (COVID-19) represented an official pandemic, because almost 125,000 cases were recognized in 118 countries and territories [1]. Because of the rapid diffusion of the COVID-19 pandemic, together with the absence of effective vaccines, the only therapeutic intervention to safeguard public health has been the adoption of strict quarantine measures. The use of containment measures for management of outbreaks is not new. Indeed, other conditions such as Severe Acute Respiratory Syndrome (SARS), Middle East Respiratory Syndrome (MERS), Ebola, H1N1 influenza, and equine influenza have required quarantines in the past.

Current and past quarantines are broadly characterized by similar measures of movement restrictions and social isolation, which vary in length (e.g., weeks, months) and degree of restrictions (e.g., closure of social activities, nationwide lockdown) as a function of pandemic diffusion, with severe consequences throughout different domains of everyday life (e.g., economic, relational, hobbies, leisure events). Despite the efficacy of movement restrictions and social isolation of confirmed and suspected cases being well-established for pandemic containment [2], the effect of quarantine on mental health is still unclear. Warnings have been issued regarding the psychopathological consequences of the recent COVID-19 disease outbreak and related quarantine measures in several letters [2,3,4,5,6] and a in qualitative review on previous outbreaks [7]. Despite the psychological burden of quarantine in terms of feelings of sadness, worries, loneliness or hypervigilance as a normal part of how human beings react to uncertain and hazardous situations, the shift towards the onset of psychopathological disorders warrants attention and investigation. 

Taken together, the diffusion of the current COVID-19 pandemic and impact of restrictive measures on psychological distress, the estimation of the burden of prolonged quarantine periods on mental health could play a key role in guiding future political choices regarding the organization of public health system. Accordingly, this study aims at conducting a systematic review of empirical research concerning the evaluation of mental health indexes (e.g., overall psychological distress, depressive, post-traumatic and anxious symptoms) during the current COVID-19 pandemic quarantine measures. Furthermore, this review aims at including previous evidence related to other pandemic infections whose management required the imposition of similar restrictive measures. This was chosen in order to generalize possible effects of quarantine measures on mental health independently of disease, length of period of containment and culture. Overall, the current systematic review aims at laying empirical foundations for planning adequate publication health interventions, especially psychological ones, considering real needs of population.

## 2. Materials and Methods

This systematic review included empirical studies that quantitively assessed mental health indexes (i.e., psychological distress, depressive, anxious, post-traumatic symptoms) during and after pandemic infections that required quarantine measures for their management. Qualitative studies and opinion papers were excluded, in accordance with the aim to quantitatively evaluate the possible effects of pandemic restrictive measures on maladaptive psychological reactions. Table 1 shows the PICO model for inclusion and exclusion criteria.

### 2.1. Search Strategy

Electronic searches were conducted of the peer-reviewed published literature on the major databases in the field of health and social sciences—PubMed, Scopus, Embase, PsycINFO, Cochrane Library and the Web of Science—with a search strategy designed to include the broadest range of relevant publications. The search was performed using MeSH terms/Keywords (depending on the database) with the same search strategy: “Quarantine” AND “Pandemic” OR “Outbreak” OR “Severe Acute Respiratory Syndrome” OR “Middle East Respiratory Syndrome” OR “COVID-19” OR “Psychological distress” OR “Anxiety” OR “Depression” OR “Emotional distress/trigger” OR “Psychiatric disorder” OR “Post-Traumatic Stress Disorder (PTSD)” OR “Adjustment disorder”. The selection of the search terms was based on the mental health literature [8]. The search was limited to English language publications, and the search period covered from January 2000 to July 2020. The starting year was 2000 because we considered the last twenty years as sufficient to cover a period that highlighted pandemic infections (e.g., MERS, SARS, H1N1, Ebola) which required the adoption of quarantine measures. The end point of the online search was July 2020 in order to include the growing evidence concerning mental health consequences of the current quarantine related to the COVID-19 pandemic. An additional examination of the reference list cited in each selected paper was also performed. Studies were excluded whenever the full text was not retrievable.

This systematic review was based on the following inclusion criteria in order to consider studies of comparable quality, as well as to sustain the reliability and validity of results: (a) studies had to report data on mental health indexes linked to epidemic infections, which required containment interventions based on quarantine; (b) only those studies were included in which valid and reliable instruments were administered, which reported the cut-off value of clinical relevance, to assess mental health impacts of quarantine; (c) studies had to be written in English.

Case reports, letters to the editor, meeting abstracts, book chapters were excluded in order to focus on data collected during and after outbreaks and related containment interventions. Reviews were considered as additional sources of information for including empirical studies within the current systematic review. Furthermore, studies carried out on health care workers were excluded because this review aimed to evaluate mental health consequences of quarantine on the general population, even though the topic has been object of a study by some authors of the same group [9]. Ultimately, qualitative studies were excluded because the objective of this review was to attempt to quantify the impact of social isolation due to pandemic infection.

### 2.2. Data Extraction

Study selection was performed by two independent reviewers with research expertise in clinical psychology (FG and RF) who assessed the relevance of studies for the objectives of this review. This first round of selection was based on the title, abstract, and keywords of each study. If the reviewers did not reach a consensus, or if the abstract did not contain sufficient information, the full text was reviewed.

**Figure 1:**
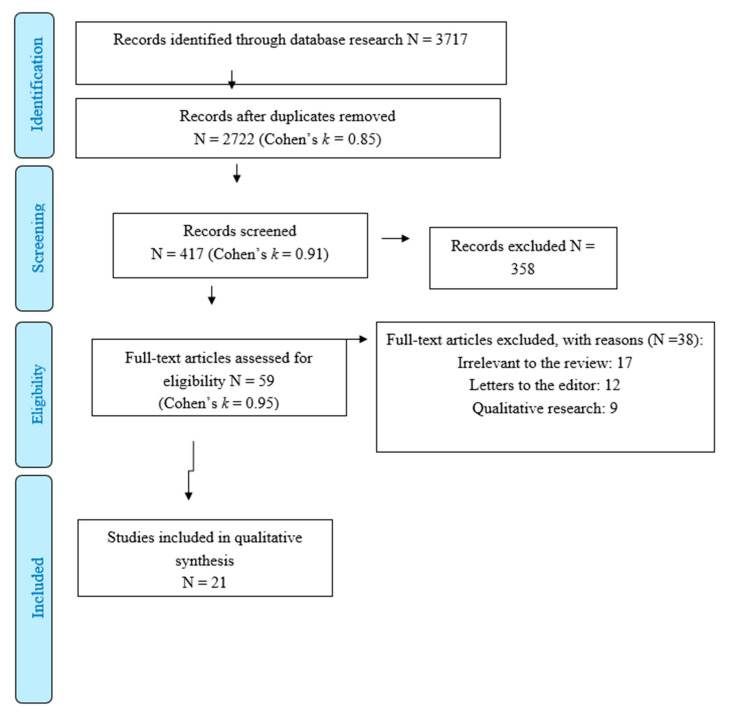
PRISMA flow diagram of literature search and selection of publications.

In the second phase (screening), full-text reports were evaluated to detect whether the studies met the inclusion criteria Figure 1. In the phase of eligibility, all full texts were retrieved, and a final check was made to exclude papers that failed to meet inclusion/exclusion criteria. A final consensus was reached to select the eventual number of studies deemed eligible.

A standardised data extraction form was prepared; data was independently extracted by two of the authors (FG and MG) and input into an initial database (N = 2.722; Cohen’s *k* = 0.85) [10]. From this initial database, FG and MG identified 417 studies (Cohen’s *k* = 0.91) that reported at least one of keywords used for the current systematic review within the title and the abstract of each manuscript. Subsequently, FG and MG excluded papers (N = 358; Cohen’s *k* = 0.95) that did not highlight a combination of keywords of this review, linking “Quarantine” or “Pandemic” or “Outbreak” or “COVID-19” AND “Psychological distress” or “Anxiety” or “Depression” or “Emotional distress/trigger” or “Psychiatric disorder” or “Post-Traumatic Stress Disorder (PTSD)” or “Adjustment disorder”. Fifty-nine studies were screened using the inclusion and exclusion criteria previously discussed. FG and MG did not reach a consensus for the inclusion of three studies, especially considering the possibility to compute the percentage of sample reporting clinically significant levels of psychological distress. Therefore, a third reviewer (MC) resolved these discrepancies [11]. Specifically, MC carefully assessed the method and results sections of each study in order to check the presence of cut-off values of instruments for the evaluation of clinically significant levels of psychological distress revealed in the sample.

### 2.3. Statistical Methods

A systematic analysis was conducted according to the Cochrane Collaboration guidelines [11] and the PRISMA Statement [12]. Considering available data, it was not possible to conduct appropriate statistical analyses linked to a meta-analytic approach [13]. However, the current review attempted to adopt a quantitative approach for aggregating results of studies. Specifically, the current systematic review used as main outcome the percentage of sample reporting clinically significant levels of psychological distress (for a description of cut-off scores (see Table S1 included as Supplementary Materials). This data was primarily reported in the Results section of each study.

SPSS 22 (IBM, Armonk, NY, USA) was used to analyze data. The analysis estimated mean, standard deviation (SD), standard error (SE) and 95% confidence interval (CI) of the percentage of sample reporting clinically significant levels of mental health outcomes (i.e., overall psychological distress, depression, anxiety and PTSD symptoms). These indexes were estimated for each outcome whenever at least three independent studies yielded data. Furthermore, in order to check for possible confounding effects of sample size and year of publications on outcomes, Spearman’s correlations (ρ) between them were performed. Furthermore, ρ was estimated between the length of quarantine period and outcomes, in order to provisionally test whether the effects of isolation on mental health depended on the period of exposure to these conditions, or alternatively, epidemic infections and related quarantine might themselves be considered triggers for psychological suffering. Bootstrap methodology (bias corrected and accelerated) was applied in computing the significance of nonparametric correlations. A total of 1000 bootstrap independent samples were used with *p* < 0.05 (2-tailed).

The analyses also compared the percentage of clinically significant distress among specific symptoms using procedures based on the Z-test [13]. The Bonferroni correction was applied when multiple comparisons were conducted. Moreover, *Z*-test procedures were used to evaluate whether other variables might affect the percentage of clinically significant psychological distress (i.e., retrospective assessment vs evaluations at the moment of epidemic infection and quarantine containment; Western culture vs Eastern culture).

### 2.4. Risk of Bias

The current systematic review assessed the quality of studies included using the rating scale developed by the National Institutes of Health for observational cohort and cross-sectional research designs [14]. This scale is composed of 14 items rated on 3 levels (i.e., Yes; No; Not applicable [NA]). “No” rating indicates the presence of possible bias. “Yes” ratings reflect methodological strengths of research. The quality of each study was independently assessed by two authors (MC and FG), who reached a high inter-rater reliability (Cohen’s *k* = 0.94). At the end of the evaluation, ratings of each study were summed within each item of risk of bias scale in order to provide a quantitative approach to the assessment of quality of studies included in the current systematic review. Given the number of studies included in this review, the total score of risk of bias scale was 294. The percentage of each rating (i.e., Yes; No; NA) on total score was computed in order to show how methodological strengths and biases were distributed across studies.

## 3. Findings

Twenty-one independent studies [15,16,17,18,19,20,21,22,23,24,25,26,27,28,29,30,31,32,33,34,35] (see Table S1 as Supplementary Material) were included for a total of 82,312 subjects. This table provides a detailed description of the characteristics of each study [15,16,17,18,19,20,21,22,23,24,25,26,27,28,29,30,31,32,33,34,35]. Table 2 summarizes aggregated results for each outcome.

First of all, up to 20% (95% CI: 14.47–27.21%) of individuals reported clinically significant levels of psychological distress during and after pandemic infections that required quarantine containment interventions. The percentage of samples reporting high levels of distress was not related to year of publication (ρ = 0.04 [−0.44–0.52]; *p* = 0.92; N = 20), sample size (ρ = −0.15 [−0.64–0.43]; *p* = 0.18; N = 20) and length of period of isolation (ρ = 0.00 [−0.50–0.50]; *p* = 1.00; N = 15). Furthermore, retrospective studies (23.33% [10.21–36.44]) showed the same results of research carried out during epidemic infections (22.21% [14.44–29.96] (Z = 0.07; *p* = 0.38). Also, the analysis did not detect an effect of culture (Western: 23.42% [16.68–30.15%]; Eastern: 20.46% [9.15–31.76%]; *Z* = 0.20; *p* = 0.39).

Considering PTSD, the results highlighted that up to 21% (95% CI: 10.95%–32.36%) of subjects reported clinically significant symptoms. The percentage of sample reporting clinically significant PTSD was not related to year of publication (ρ = −0.11 [−0.77–0.63]; *p* = 0.76; N = 10), sample size (ρ = −0.39 [−0.83–0.33]; *p* = 0.26; N = 10) and length of period of isolation (ρ = 0.62 [−0.45–1.00]; *p* = 0.19; N = 6).

Similar findings were extended to depressive symptoms. Specifically, studies found that 22.69% (95% CI: 13.04%–32.33%) of participants highlighted clinically relevant symptoms of depression. The percentage of samples reporting high levels of depressive symptoms was independent of year of publication (ρ = 0.02 [−0.59–0.57]; *p* = 0.95; N = 12), sample size (ρ = −0.03 [−0.76–0.80]; *p* = 0.91; N = 12) and length of quarantine (ρ = −0.33 [−0.97–0.95]; *p* = 0.42; N = 6). The percentage of clinically significant symptoms of PTSD was not statistically different from the depressive ones (Z= −0.05; *p* = 0.40).

The analysis revealed lower rates (16.16%; 95% CI: 8.20%–24.12%) of clinically relevant symptoms of anxiety than PTSD and depression symptoms, albeit not statistically different (PTSD: Z = −32; *p* = 0.38; depressive: Z= −0.05; *p* = 0.40). The percentage of clinically significant symptoms of anxiety were not affected by the year of publication (ρ = 0.56 [−0.07–0.91]; *p* = 0.07; N = 11), sample size (ρ = 0.30 [−0.38–0.80]; *p* = 0.37; N = 11) and length of period of isolation (ρ = −0.28 [−1.00–0.65]; *p* = 0.54; N = 7).

Only two studies [28,35] investigated the role of relational style as protective factors for psychological distress. On the one hand, Moccia and colleagues [28] showed that Attachment Style Questionnaire subscales, namely “Confidence” (OR: 0.92; *p* = 0.039) and “Discomfort with closeness” (OR: 0.94; *p* = 0.023), were modestly associated with mental health during the COVID-19 quarantine. On the other hand, Germani and colleagues [35] found that horizontal collectivism—in which people see themselves as being similar to others and emphasis is placed on common goals with others, interdependence, and sociability—was a significant protective factor for higher psychological burden (*p* < 0.001) during the same pandemic infection. Additional studies estimated the role of gender on the development of clinically significant psychiatric symptoms [26,27,31] highlighting that females reported greater risk for psychological distress than male, with one exception [34].

Risk of bias assessment Table 3 showed a total score of overall strengths of studies included equal to 107 (36.3%). The facet reflecting overall biases highlighted a score of 111 (37.7%). Therefore, methodological strengths and biases were equally distributed across studies included in the current system review. The most recurrent weakness of studies referred to the absence of multiple longitudinal assessments (21 studies) and the lack of adequate control of possible confounding factors on outcomes (20 studies).

## 4. Discussion

The current systematic review sought to empirically estimate the impact of epidemic infections and related quarantine containment measures on mental health and psychological distress [36]. Specifically, this study attempted to lay the foundations for an appropriate response of mental health services. Accordingly, the current review aggregated results from prior studies that investigated this topic during similar epidemic infections, albeit significantly less widespread, together with provisional data collected during the current COVID-19 outbreak.

Overall, empirical findings suggested that at least one out of every five people reported clinically significant psychological distress, independently of the length of isolation and culture. This finding is fully in line with other empirical studies that demonstrated how medical condition-related isolation significantly predicted the onset of psychological distress [37]. In addition to the adverse effects of isolation, the psychological distress linked to epidemic infections is largely in keeping with the well-recognized dysfunctional cognitive and emotional mechanisms underlying the fear of contamination [38]. With respect to the onset of specific symptoms, the analysis showed that the most recurrent clinical conditions associated with epidemic infections referred to PTSD and depression. Specifically, up to 20% of individuals reported the onset of clinically significant symptoms of such conditions. Since January 2020 (the time of declaration of COVID-19 in China), a sentiment analysis of Chinese social media showed an increase in negative emotions (anxiety and depression) and a decrease in life satisfaction [39].

Considering PTSD symptoms, this evidence might be related to fact that an epidemic infection represents a direct or indirect exposure to a threatened (potential) death, which is considered the core diagnostic criterion for PTSD [8]. Additionally, the high occurrence rates of clinically significant levels of depression might be linked to the well-established effects of social isolation and relational deprivation on emotional and cognitive functioning [40,41]. The high occurrence of generalized anxiety symptoms, albeit less pronounced than the other conditions, might be associated with a heightened vigilance [42] and worry [43] towards threats related to diffusion trends of epidemic infections and their consequences on everyday life [44]. According to this evidence, it is plausible to expect that at the end of the current COVID-19 pandemic, one out of every five people might develop clinically significant psychological distress. Particularly, the recurrent conditions might be PTSD, depression and (less) generalized anxiety.

Further provisional evidence suggested the existence of risk or buffering factors for developing psychopathology, which should be carefully considered in order to provide effective psychological interventions. From the current review, some evidence arose towards a protective role for factors such as secure or avoidant attachment style [28] and social openness (sharing common goals with others, interdependence, and sociability) [35]. Conversely, female gender, negative affect, and detachment [27], dysfunctional personality traits or temperament (negative affectivity, detachment and disinhibition) [28,32], severe property damage and low self-perceived health condition [24], younger age with/without chronic disease [17,30] or feeling extreme fear [33] seem to be predictive of the worst outcomes in terms of mental health. Current findings are consistent with the published literature on trait characteristics and mental health outcomes, which found that personality dimensions such as neuroticism, female gender, younger age and chronic disease are positively associated with poorer mental health outcomes [45,46,47]. Moreover, state variables such as fear of infection during pandemic are associated with elevated levels of psychological distress [7,48]. Furthermore, the length of quarantine was not associated to the mental health outcome. This important aspect remarks the importance of studying which factors (fear of contagion? personality characteristics? psychopathological diathesis) candidate individuals to a worst outcome in term of mental health.

Examining such symptomatology and the context of its development, mental health professionals should provide tailored assessment and interventions for distressing and post-traumatic reactions.

In the context of COVID-19, psychological assessment and monitoring should include queries about COVID-19–related stressors (such as exposure to infected sources, infected family members, loss of loved ones, and physical distancing), secondary psychological consequences (financial loss, depression, anxiety, psychosomatic preoccupations, sleep disorders, increased substance use, familial conflicts and/or domestic violence), and indicators of previous vulnerability conditions (such as pre-existing physical or psychological conditions). Psychoeducation or cognitive behavioral techniques may be beneficial for some patients; others will benefit from formal mental health evaluation and care [49].

Despite this systemic review showing a clear scenario concerning psychological effects of epidemic infections and related quarantine containment interventions, certain limitations must be discussed. First, all studies were based on the administration of self-report measures that might produce a portion of false-positive cases. Therefore, the current findings should be confirmed using adequate structured clinical interviews (e.g., Structured clinical interview for DSM-5 disorders: Clinician version) [17]. Second, all studies included were cross-sectional naturalistic research designs. This did not allow investigators to reach the definitive conclusion that the event of epidemic infection and related quarantine interventions are the undoubted primary causes of the onset of psychological distress. Hence, empirical research should identify concurrent risk factors that facilitate the development and maintenance of these clinical conditions. The studies cover a range of different viruses/conditions and a range of participants, including those who had the condition, those who did not have the condition but family members who did, and those who did not have the condition but were quarantined. It is possible that different types of exposures will produce very different types of psychological reactions and produce different rates of distress/psychopathology [9]. Other factors (e.g., socioeconomic status, access to medical care, chronic disorders etc.) would also affect psychological distress and lead to different rates of psychological distress. Ultimately, the absence of multiple longitudinal assessments did not allow the precise evaluation of the course of psychological distress after epidemic infections and quarantining containment procedures. Furthermore, the lack of evaluation of incidence rates of each psychological condition before the onset of epidemic infections limited the possibility to draw definitive conclusions concerning the extent of impact of such phenomena on development of psychological distress. At the present moment, there are few studies to examine these different critical issues and produce robust and replicable estimates of psychological distress. However, this historical period represents a matchless opportunity to study the clinical psychological burden of quarantine both for the prolonged period and for the worldwide diffusion of social isolation. Last but not least, the wide use of surveys with participation on a voluntary basis adds an important risk of bias. We need to study the psychological consequences of quarantine addressing the way people cope with social isolation, the predictors of maladaptive functioning, the role of pre-COVID-19 personality and mental health issues. Special attention should be paid to people after COVID-19 hospitalization in order to test the burden in terms of PTSD, anxiety and/or depression.

Despite these limitations, this is the first quantitative systematic review that provisionally estimates the extent of psychological distress associated with past and current epidemic infections and related quarantine interventions. Results suggest that almost one out of every five people is at risk of development of clinically significant psychological distress during and after epidemic infections. Accordingly, mental health professionals should prepare to address a possible pandemic psychological suffering linked to the current COVID-19 pandemic infection.

## Figures and Tables

**Table 1:** PICO model for inclusion and exclusion criteria.

Model Category	Inclusion Criteria	Exclusion Criteria
Population	General population, students	Health care workers
Intervention	Direct or indirect exposure to quarantine measures for management of pandemics	Opinion papers on psychological effects of quarantine measures
Comparison	−	−
Outcomes	Quantitative evaluation of overall psychological distress and severity of depressive, anxious and post-traumatic symptoms	Qualitative assessment of mental health indexes

**Table 2:** Summary of aggregated results.

Outcome	Number of Studies (Total Sample)	% of Sample Reported Clinically Significant Levels (95% CI)	Number of Individuals Reported Clinically Significant Levels
Overall psychological distress	21 (82,312)	20.84% (14.47–27.21)	17,154
PTSD symptoms	10 (7725)	21.65% (10.95–32.36)	1672
Depressive symptoms	13 (74,407)	22.69% (13.04–32.33)	16,883
Anxious symptoms	11 (73,458)	16.16% (8.20–24.12)	11,871

**Table 3:** Assessment of risk of bias (N = 21).

Criteria	Yes	No	NA
1. Was the research question or objective in this paper clearly stated?	19	2	0
2. Was the study population clearly specified and defined?	17	4	0
3. Was the participation rate of eligible persons at least 50%?	8	6	7
4. Were all the subjects selected or recruited from the same or similar populations (including the same time period)? Were inclusion and exclusion criteria for being in the study prespecified and applied uniformly to all participants?	20	1	0
5. Was a sample size justification, power description, or variance and effect estimates provided?	4	17	0
6. For the analyses in this paper, were the exposure(s) of interest measured prior to the outcome(s) being measured?	0	21	0
7. Was the timeframe sufficient so that one could reasonably expect to see an association between exposure and outcome if it existed?	1	20	0
8. For exposures that can vary in amount or level, did the study examine different levels of the exposure as related to the outcome (e.g., categories of exposure, or exposure measured as continuous variable)?	0	0	21
9. Were the exposure measures (independent variables) clearly defined, valid, reliable, and implemented consistently across all study participants?	21	0	0
10. Was the exposure(s) assessed more than once over time?	0	0	21
11. Were the outcome measures (dependent variables) clearly defined, valid, reliable, and implemented consistently across all study participants?	14	6	1
12. Were the outcome assessors blinded to the exposure status of participants?	1	14	6
13. Was loss to follow-up after baseline 20% or less?	1	0	20
14. Were key potential confounding variables measured and adjusted statistically for their impact on the relationship between exposure(s) and outcome(s)?	1	20	0
Total score:	107	111	76

NA = Not Applicable.

## Data Availability

The data presented in this study are available on request from the corresponding author. The data are not publicly available due to the decision of the Authors.

## Supplementary Materials

The following are available online at https://www.mdpi.com/2227-9032/9/1/101/s1, Table S1: Overview of the selected studies.
