# Supplementary material for: What Will Be the Impact of the COVID-19 Quarantine on Psychological Distress? Considerations Based on a Systematic Review of Pandemic Outbreaks
Source: Healthcare (Basel). 2021 Jan 19;9(1):101. doi: 10.3390/healthcare9010101 (PMC7835976; doi:10.3390/healthcare9010101)
Supplement: Supplementary file 1 [file healthcare-09-00101-s001.pdf]

Table S1. Overview of the selected studies.

Study	Sample description	Country	Disease	Study design	Quarantine length	Assessment tools	Outcome measure	% of clinical distress	Other significant findings	Note
Hawryluck et al., 2004 [15]	*N* = 129<br>Gender distribution non reported	Canada	SARS	Cross-sectional General population survey	Median 10 days	IES-R (cut-off ≥ 20)<br>CES-D (cut-off ≥ 16)	PTSD symptoms<br>Depressive symptoms	Overall 30.05%<br>PTSD 28.9%<br>Depression 31.2%	The duration of quarantine was significantly related to increased PTSD and depressive symptoms	Responders completed the survey at the end of quarantine and within the pandemic
Reynolds et al., 2008 [16]	*N*=1057<br>(37% M; mean age 49.2)	Canada	SARS	Cross-section General population survey	8 days	IES-R (cut-off ≥ 20)	PTSD symptoms	PTSD 14.6%	Health-care workers experienced more severe symptoms of PTSD ($p < .001$)	Responders completed the survey during quarantine and within the pandemic
Taylor et al., 2008 [17]	*N*=2760<br>(15% M; mean age not reported)	Australia	Equine influenza	Cross-sectional Retrospective General population survey	Not reported	K10 (cut-off ≥ 30)	Anxiety and Depression	Anxiety and depression 14%	Individuals who lived in a high-risk infection (red) zone were at much greater risk of high psychological distress.<br>Younger people, and those with lower levels of formal educational qualifications were at greater risk of high psychological distress	Responders were quarantined during the pandemic
Wang et al., 2011 [18]	*N* = 419<br>Quarantined *N*= 176 Nonquarantined *N* = 243	China	H1N1	Cross-sectional case-control studies	7 days	IES-R (cut-off ≥ 20)<br>SRQ-20 (cut-off ≥ 7)	PTSD symptoms<br>General mental health	Overall 12.9%<br>Overall PTSD 14.3%<br>PTSD Quarantined 10.8%	No significant differences for positive screening measures between quarantined and nonquarantined students	The survey was completed for all participants at the end of the quarantine period during the pandemic.

								PTSD<br>Not quarantined<br>16.87%<br><br>Overall general mental health<br>11.45%<br><br>General mental health quarantined<br>7.95%<br><br>General mental health<br>Not quarantined<br>14%		
Sprang & Silman, 2013 [19]	*N*= 398<br>(22% M; mean age = 37)	USA	H1N1	Cross-sectional<br>Retrospective<br>General population survey	Not reported	PCL-C parent<br>(cut-off ≥ 25)<br><br>PTSD-RI Child<br>(cut-off not reported)	PTSD symptoms	PTSD<br>25%	5.8% of parents scored above 30 on the PCL-C, indicating that the diagnostic threshold for PTSD was met. Children who experienced isolation or quarantine were more likely to meet the clinical cutoff score for PTSD (30%) than those who had not been in isolation or quarantine	Responders were quarantined during the pandemic
Jeong et al., 2016 [20]	*N*=1692;<br><br>*N* = 36 MERS cases<br>(50% M; mean age = 52.3)	Korea	MERS	Case-control<br>(MERS cases vs isolated)<br>Isolated people survey	21 days	STAXI<br>(cut-off ≥ 14)<br><br>GAD-7<br>(cut-off ≥ 10)	Anger<br><br>Anxiety symptoms	Overall<br>9.02<br><br>Anger during isolation<br>17.4%	Patients with MERS had significant high rates of clinical anger and anxious during the isolation period:	Responders were assessed during the isolation period and four to six months after removal from isolation.

	N= 1656 isolated people<br>(43% M; mean age = 43.9)							Anger after isolation 6.9%<br><br>Anxiety during isolation 8.4%<br><br>Anxiety after isolation 3.4%	MERS $_{anger}$ = 52.8% (95% CI: 36.5 - 69.1%)<br><br>Isolated $_{anger}$ = 16.6% (95% CI: 14.8 -18.4%)<br><br>MERS $_{anxiety}$ = 47.2% (95% CI: 30.9 - 63.5%)<br><br>Isolated $_{anxiety}$ = 7.6% (95% CI: 6.3 - 8.9%)<br><br>These differences were replicated after removal from isolation<br><br>MERS $_{anger}$ = 30.6% (95% CI: 15.6 - 45.7%)<br><br>Isolated $_{anger}$ = 6.4% (95% CI: 5.2 - 7.6%)<br><br>MERS $_{anxiety}$ = 19.4% (95% CI: 6.5 - 32.3%)<br><br>Isolated $_{anxiety}$ = 3.0% (95% CI: 2.2 - 3.9%)	
Jalloh et al., 2018 [21]	*N* = 3564<br>(50% M; median age = 35)	Sierra Leone	Ebola	Cross-sectional Retrospective General population survey	21 days	IES-6<br>(cut-off ≥ 1.09 mean item equivalent to 24 on IES-R)<br><br>PHQ-4	PTSD symptoms<br><br>Depressive and anxious symptoms	Overall 11%<br><br>PTSD 16%<br><br>Depression 6%	Those participants who knew someone quarantined due to Ebola exposure alone were more likely to report symptoms of	Responders were not directly quarantined

						(cut-off ≥ 6)			anxiety and depression (OR = 2.3; 95%CI: 1.7-2.9, *p* < .001) and PTSD (OR = 2.0; 95%CI: 1.5 - 2.8, *p* < .001)<br><br>Respondents who had both experiences (that is, they knew at least one person who died from Ebola and someone quarantined) were also more likely to report symptoms of anxiety and depression (OR = 1.8; 95%CI: 1.5 - 2.2, *p* < .001) and PTSD (OR = 2.3 95%CI 1.8 – 2.8; p < .001)	
Kim et al., 2018 [22]	*N*= 27 (37% M; mean age = 41.15)	Korea	MERS	Cross-sectional<br><br>Assessment of hospitalized individuals with confirmed (*N* = 18) and suspected (*N* = 9) MERS	Not reported	IES-R (cut-off not reported)<br><br>KNHANES-short form (cut-off not reported)<br><br>PHQ-9 (cut-off ≥ 10)	PTSD symptoms<br><br>Levels of stress<br><br>Depressive symptoms	PTSD Not reported<br><br>Levels of stress Not reported<br><br>Depressive symptoms 40.7%	Confirmed Mers:17 (70.8%) exhibited psychiatric symptoms and 10 (41.7%) received a psychiatric diagnosis.<br><br>Suspected MERS did not exhibit psychiatric symptoms	Participants were assessed during MERS pandemic (3 months) and therapeutic isolation
Lee et al., 2018 [23]	*N*= 432<br>*N*= 359 hospital workers (18.1% M; mean age not reported)	Korea	SARS	Cross-sectional Hospital workers survey and clinical assessment procedures of patients with SARS	Not reported	IES-R Hospital workers (cut-off ≥ 25)<br><br>MINI Inpatients Depression	PTSD symptoms<br><br>Depressive symptoms<br><br>Anxiety symptoms	Overall 25.6%<br><br>PTSD Hospital workers 51.5%	The healthcare workers who performed MERS-related tasks had significantly higher total IES-R scores (*t* =3.89, *p* <.001)	The initial sample of hospital workers were not quarantined. A second survey assessed PTSD symptoms among hospital workers (N=77; quarantined

	N=73 hemodialysis patients (56.2% M; mean age = 61.3 (Sars, South Korea)					(cut-off ≥ 5)<br>Anxiety<br>(cut-off ≥ 3)<br><br>HADS<br>Inpatients<br>Depression<br>(cut-off ≥ 8)<br>Anxiety<br>(cut-off ≥ 8)		Depressive symptoms inpatients 10.3%<br><br>Anxiety symptoms inpatients 11%	and sub-scores, including hyperarousal ($t = 3.535$, $p < .001$), avoidance ($t = 3.573$, $p < .001$), intrusion ($t = 3.756$, $p < .001$), and sleep and numbness ($t = 3.583$, $p < .001$)	$N = 23$; not quarantined $N = 54$). However, the study did not report the % of sample which exceeded the IES-R cut-off score
Lei et al., 2020 [24]	*N*=1593 (38.7% M)	China	COVID-19	Cross-section study	30 days	SAS<br>(cut-off ≥ 50)<br><br>SDS<br>(cut-off ≥ .50)	Depressive symptoms<br><br>Anxiety symptoms	Depression 7.3%<br><br>Anxiety 4.5%	Anxiety in affected was predicted by average household income (p = 0.028), self-perceived health condition (p < 0.001), property damage (p = 0.003); in unaffected group by divorced/widowed (p = 0.001), self-evaluated level of knowledge (p = 0.032) and self-perceived health (p= 0.001).<br>Depression in affected group predicted by education level (p = 0.015), self-perceived health condition (p < 0.001), property damage (p = 0.002); in unaffected group by divorced/widowed (p < 0.001), self-perceived health condition (p < 0.001), being worried	The study assessed several psychosocial risk for the onset of psychiatric symptoms linked to quarantine.<br><br>No subgroup analyses were conducted considering different levels of exposure to illness

								about being infected (p = 0.006) and presence of psychological support (p =0.043).	
Li et al., 2020 [25]	*N*= 5033 (33.3% M)	China	COVID-19	Cross-sectional General population survey	Not reported	GAD-7 (cut-off ≥ 8)<br><br>PHQ-9 (cut-off ≥ 8)	Anxiety<br><br>Depression	Anxiety 20.4%<br><br>Depression 20.4%	Anxiety and/or depression was significantly associated with time spent on COVID-19-related news per day (p<0.001). Anxiety was associated with psychological stressors such as “I worry about myself and my loved ones being infected by COVID-19” (OR=1.95, 95% CI: 1.54-2.49), “I worry about my income, job, study or ability to pay the loan being affected” (OR=1.38, 95% CI: 1.13-1.68), and “Home quarantine causes great inconvenience to my daily life” (OR=1.31, 95% CI: 1.04-1.64). The same psychological stressors were associated with depression (respectively OR=1.24, 95% CI: 1.04-1.50; OR=1.58, 95% CI: 1.35-1.86; OR=1.42, 95% CI: 1.18- 1.70)	The study evaluated different content of anxiety and depression symptoms linked to consequences of specific stressors.<br>The absence of longitudinal data did not allow the investigators to assess the temporal stability of risk factors for the development of psychiatric symptoms

Liu et al., 2020 [26]	*N* = 217 (41.5% M; mean age 21.7)	China	COVID-19	Cross-sectional student population survey	30 days	GAD-7 (cut-off ≥ 8)<br>PHQ-9 (cut-off ≥ 8)	Depression<br>Anxiety	Depression 35.5%<br>Anxiety 22.1%	Prevalence of depression and anxiety were not significantly different in students according to gender (p = 0.155), Geographical location (p = 0.356) and grade (p = 0.097).	The study assessed a high risk population for developing psychiatric conditions as a consequence of quarantine.<br>The number of participants of study was too small to generalize results to other student populations
Mazza et al., 2020 [27]	*N*= 2766 (28.3% M; mean age 32.94)	Italy	COVID-19	Cross-sectional General population survey	14 days	DASS-21 depression (cut-off ≥ 21)<br>DASS-21 anxiety (cut-off ≥ 15)<br>DASS-21 stress (cut-off ≥ 27)	Depression symptoms<br>Anxiety symptoms<br>Stress symptoms	Depression 32.8%<br>Anxiety 19.0%<br>Stress 29.2%	Stress was associated with female gender (p<0.001), negative affect, (p<0.001), detachment (p<0.001), acquaintance infected (p<0.001). Depression with female gender (p<0.001), negative affect (p<0.001), detachment (p<0.001), having an acquaintance infected (p<0.001) history of stressful situations (p = 0.008), medical problems (p= .047). Anxiety was associated with female gender (p<0.001), negative affect (p<0.001), detachment (p<0.001), history of stressful situations (p = 0.008), medical problems (p = 0.001), and a	This longitudinal study evaluated the impact of personality as a relevant risk factor for the onset of psychiatric symptoms.<br>The study also included the assessment of different levels of exposure to illness

									family member infected (p<0.001).	
Moccia et al., 2020 [28]	*N* = 500 (40.4% M)	Italy	COVID-19	Cross-sectional General population survey	30 days	K10 (cut-off ≥ 19)	Anxiety and Depression	Anxiety and depression 18.6%	Cyclothymic (p < 0.001), depressive (0.001), anxious temperament (p = 0.002) and sub-item "Need for Approval" (p = 0.01) were risk factors for moderate-to-severe psychological distress; sub-item "Confidence" (p = 0.002) and sub-item "Discomfort with closeness" (p = 0.001) of Attachment Style Questionnaire were protective factors.	The study showed that interpersonal styles were risk and protective factors for the development of psychiatric symptoms. The online survey lasted only four days.
Odriozola-González et al., 2020 [29]	*N*= 2530 (33.9% M)	Spain	COVID-19	Cross-sectional University survey	10 days	DASS-21 (cut-off: not reported)<br>IES (cut-off ≥26)	Depression Anxiety Stress<br>PTSD symptoms	Depression 34.19%<br>Anxiety 21.34%<br>Stress 28.14%<br>PTSD symptoms 12.5%	Students from Arts & Humanities and Social Sciences & Law showed higher scores related to anxiety, depression, stress and impact of event with respect to students from Engineering & Architecture. University staff presented lower scores in all measures compared to students, who seemed to have suffered an important psychological impact during the first weeks of the COVID-19 lockdown.	The study compared the psychological impact of outbreak between students from different departments and staff of the university. The study did not include the evaluation-specific factors that could explain the difference in levels of severity of psychiatric symptoms among these groups

Ozamiz-Etxebarria et al., 2020 [30]	N = 976 (18.9% M)	Spain	COVID-19	Cross-sectional General population survey	Not reported	DASS-21 (cut-off: not reported)	Depression/Anxiety Stress	Depression 22.0% Anxiety 28.8% Stress 22.0%	Younger individuals with chronic diseases reported more symptoms than the rest of the population. The study also detected higher levels of symptoms after the stay-at-home order was issued. Such symptoms are predicted to increase as the confinement continues.	The study did not consider psychosocial risk factors that were involved in explaining severity of psychiatric symptoms. The study recruited participant from a specific region of Spain
Qiu et al., 2020 [31]	N = 52730 (35.2% M)	China	COVID-19	Cross-section General population survey	21 days	CPDI (cut-off $\geq$ 52)	Anxiety and depression symptoms, together with related behaviors	Anxiety and Depression 35%	Female respondents showed significantly higher psychological distress than their male counterparts ($p < .001$). Individuals between 18 and 30 years of age or above 60 presented the highest CPDI scores	Responders completed the survey during the quarantine
Somma et al., 2020 [32]	N= 1043 (18.5% M)	Italy	COVID-19	Cross-section General population survey	10 days	SDQ EPS (cut-off $\geq$ 7)	Emotional and behavioral problems	Overall 13.2%	Negative affectivity (t(1041) = 19.02, Cohen's d = 1.18) and detachment (t(1041)=13.32, Cohen's d = 0.83) represented relevant risk factors for reduced emotional well-being	The study showed that maladaptive personality traits were involved in explaining the onset of psychological distress during quarantine. The characteristics of the sample did not allow the investigators to generalize results to the Italian population

Tang et al., 2020 [33]	*N*=2485 (39.2% M)	China	COVID-19	Cross-sectional Students population survey	30 days	PCL-C (cut-off ≥ 38)<br>PHQ-9 (cut-off ≥ 10)	PTSD symptoms<br>Depressive symptoms	PTSD 2.7%<br>Depression 9.0%	Feeling extreme fear was the most significant predictor for both depression (, $p<0.001$) and PTSD ($p<0.001$), followed by short sleep duration ($p<0.001$), living in the worst-hit areas ($p<0.001$); Sleep duration was observed to be a mediator between number of exposures and PTSD (z=0.104, 95% CI: 0.016, 0.204), or depression (z= .065, 95% CI: 0.010, 0.126)	The study showed a key role of neurovegetative alterations on the development of psychiatric symptoms during quarantine.<br>The study recruited a sample composed of students. Therefore, this characteristics of sample did not allow the investigators to generalize the results
Fawaz et al., 2020 [34]	*N* = 950 (69.3% M)	Lebanon	COVID-19	Longitudinal survey on general population	30 days	PCL-C (cut-off ≥ 3)	PTSD symptoms	37.72%	No difference in the prevalence of PTSD symptoms among genders ($p = .07$), among occupations (health care worker or not, $p = .34$), age ($p = .15$) and leaving home during quarantine or not ($p = .77$), but the possible sources of exposure to COVID-19 ($p = .02$). Gender, age, occupation, potential sources of exposure and leaving home or not were not predictors of PTSD.	This study was carried out a pre- post-test research design. Furthermore, the investigators included the evaluation of different levels of exposure to illness. However, the study did not consider relevant psychosocial risk factors for the development of psychiatric symptoms

Germani et al., 2020 [35]	*N*= 1011 emerging adults (28.7% M)	Italy	COVID-19	Cross-sectional General population survey	STAI-Y (cut-off ≥ 40)<br>PSS (cut-off ≥ 14)	Anxiety<br>Stress	Not reported	State anxiety (STAI-Y, mean ± SD: 48.56 ± 12.73) and stress levels (PSS, mean ± SD: 21.59 ± 7.16) were above the normal cut-off. Collectivistic orientation was related to higher perceived risks of infection (horizontal collectivism with general concern: coeff = 0.18; with personal concern: coeff = 0.12; with relatives/others concern: coeff = 0.13, all p<0.001; vertical collectivism with general concern: coeff = 0.20; with personal concern: coeff = 0.23; with relatives/others concern: coeff = 0.18, all p<0.001) and predicted lower psychological maladjustment, controlling for socio-demographic variables (horizontal collectivism: B = -0.24, -0.3 - -0.18 95% CI, p<0.001).	The study assessed the role of cultural and several psychosocial factors on the severity of psychiatric symptoms during quarantine. The convenience sampling method did not allow the investigators to generalize results

CES-D = Center for Epidemiologic Studies—Depression Scale; CPD = COVID-19 Peritraumatic Distress Index; GAD-7= Generalized Anxiety Disorder Scale; HADS = Hospital Anxiety and Depression Scale; IES-6: Impact of Event Scale-6; IES-R: Impact of Event Scale-Revised; K-10 = Kessler 10; KANES: Korean National Health and Nutrition Examination Survey; M= Men; MERS: Middle East Respiratory Syndrome; MINI = Mini International Neuropsychiatric Interview; PCL-C = PTSD Check List - Civilian Version; PHQ-4,9 = Patient Health Questionnaire-4,9; PSS: Perceived Stress Scale; PTSD= post-traumatic stress disorder; PTSD-RI = PTSD Check List - Civilian Version; SDQ-EPS = Strengths and Difficulties Questionnaire Emotional Problems scale; SRQ-20 = Self-Report Questionnaire STAXI = State-Trait Anger Expression Inventory.
